# Ethnobotanical study of medicinal plants in the Hawassa Zuria District, Sidama zone, Southern Ethiopia

**DOI:** 10.1186/s13002-019-0302-7

**Published:** 2019-05-24

**Authors:** Banchiamlak Nigussie Tefera, Young-Dong Kim

**Affiliations:** 1 Department of Life Science and Multidisciplinary Genome Institute, Hallym University, Chuncheon, 24252 Republic of Korea; 2 Ethiopian Institute of Agricultural Research, P.O. Box 2003, Addis Ababa, Ethiopia

**Keywords:** Medicinal plants, Traditional knowledge, Sidama people, Hawassa Zuria district, Ethiopia

## Abstract

**Background:**

Ethiopia is one of the species-rich countries in the world and the center of origin with regard to the diversity of many plant species. Ethnobotanical studies are vital to investigate these diverse biological resources for medicinal purposes. The aim of this study was to document the indigenous knowledge of the Sidama people regarding the use of medicinal plants to treat human and livestock diseases in the Hawassa Zuria district of Southern Ethiopia.

**Methods:**

A total of 150 informants (118 men and 32 women) were selected to collect ethnobotanical information from ten kebeles by means of a stratified purposive sampling method. Among the informants, 30 key informants were selected purposefully. Ethnobotanical data were collected through semi-structured interviews and group discussions and were analyzed by descriptive statistics, informant consensus factor (ICF), fidelity level (FL), and ranking methods.

**Results:**

A total of 105 medicinal plants distributed across 52 families and 96 genera were collected. Fabaceae (11 species) was represented by the highest number of plant species, followed by Lamiaceae (7 species). Herbs (34%) were the dominant growth habits, followed by trees (33%). Leaves (56%) were the dominant plant part used in the preparation of remedies, followed by fruit (15%). The most common method of remedy preparation was grinding (39%) followed by chewing and boiling (11% each). Oral (74%) was the major routes of administration, followed by dermal (20%). There was a significant knowledge difference (*p* < 0.05) between social groups regarding the use of traditional medicinal plants. Insects and ectoparasites disease category (0.95) had higher informant consensus factor value followed by fever disease category (0.91). *Eucalyptus globulus* Labill. (100%) had a higher fidelity level to treat stomachache, followed by *Ensete ventricosum* (Welw.) Cheesman. (87.27%) to treat placenta delay. *Ensete ventricosum* (total score = 73) was ranked highest as the most preferable medicinal plant for various purposes by local people, followed by *Olea welwitschii* (Knobl.) Gilg (total score = 72).

**Conclusion:**

The present study revealed the existence of indigenous knowledge of medicinal plants to treat human and livestock ailments. However, agricultural expansion, firewood collection, environmental degradation, and deforestation are the main threats to medicinal plants. Therefore, there should be mentoring for the local people in the study area to conserve their indigenous knowledge resources and prevent the extinction of medicinal plants.

**Electronic supplementary material:**

The online version of this article (10.1186/s13002-019-0302-7) contains supplementary material, which is available to authorized users.

## Introduction

Human beings have depended on nature for their simple requirements as being the source of medicines, shelters, food, fragrances, clothing, flavors, fertilizers, and means of transportation throughout their lives. Plants have been used for medicinal purposes since long before the prehistoric period [1].

Medicinal plants have made a significant contribution to the primary healthcare of people around the world. Population increases, inadequate supplies of drugs, the prohibitive cost of treatments, side effects of several synthetic drugs, and the development of drug resistance to infectious diseases have led to the increasing use of plant materials as a source of medicine for a wide variety of human ailments. Recently, the WHO estimated that 80% of people worldwide rely on herbal medicines for some aspects of their primary healthcare needs. According to the WHO, around 21,000 plant species can potentially be used as medicinal plants [2].

Africa has rich resources of medicinal plant species. Ethiopia is believed to be home for about 6500 to 7000 species, with approximately 12% of these endemic [3]. In Ethiopia, approximately 80% of humans and 90% of the livestock population rely on traditional medicinal plants to cure different ailments [4] due to difficulties in accessing modern health facilities, the cultural acceptability of healers, and low cost of traditional medicine [5].

Southern Ethiopia is the main homeland of numerous ethnicities, containing more than 45 indigenous ethnic groups who speak at least 12 languages from four linguistic families [6]. The Sidama ethnic group (19.38%) is the predominant group in the Southern Nations Nationalities and Peoples Region. They number about 4.8 million, of whom 3.9 are urban inhabitants. The main spoken language is Sidamegna (18%) from the Cushitic linguistic family [7]. The daily lives of the Sidama peoples depend on agriculture. Ensete (*Ensete ventricosum* (Welw.) Cheesman.), also known locally as the *wesse* plant, is an important staple food. Coffee (*Coffee Arabica* L.) is the most important source of income, and the Sidama zone is the major contributor to coffee production. The Hawassa Zuria district is well known as a maize (*Zea may*s L.) growing district, with other crops also grown. The people raise cattle, and there is high value attached to livestock by the Sidama. The number of cattle owned is a good indicator of wealth, and popularity increases for farmers who own more cattle. The zone is also rich in water resources, which are underutilized.

Greater numbers of medicinal plants are found in the south and southwestern parts of Ethiopia due to the high biological and cultural diversity in these regions [3, 8]. Thus far, 1000 identified medicinal plant species have been reported among Ethiopian flora, but others remain not yet identified. There are approximately 887 medicinal plant species that are currently used by the Ethiopian people. Nearly 300 of these are frequently mentioned in many sources. The majority of medicinal plants are herbs, followed by shrubs and trees [9]. Most of the medicinal plant species are found in wild forests [10]. Over 40% of medicinal plant species have enormous socio-economic value in Ethiopia, and these require further investigations [11].

Ethnobotanical studies documented in Southern Ethiopia have studied the following: the Amaro district [12], the Benna Tsemay district [13], Burji district [14], Cheha district [15], Kembatta ethnic group [16], Konsso ethnicity [17], the Konta special woreda [18], the Lemo district [19], Maale and Ari [20], the Wolaita zone [21], the Wonago woreda [3], and the Wolaita zone [22]. However, there is still limited ethnobotanical documentation on medicinal plants and relatively few phytochemical analyses of documented medicinal plants. Ethnobotanical studies of medicinal plants conducted in the Sidama zone of southern Ethiopia have focused on the Boricha district [23], the Dale district [24], Hawassa city [25], the Bensa district [26], and Wondo genet [27].

The greater concentration of medicinal plants is found in the south and southwestern parts of the country in keeping with the concentration of biological and cultural diversity [28]. This indicates that there is high traditional medicinal plant knowledge in the southern part of Ethiopia, but the indigenous knowledge has not been systematically documented in the region. Particularly, there is no ethnobotanical study in the current study area of the Hawassa Zuria district. In addition, indigenous knowledge is disappearing due to a lack of written documents about medicinal plants, the deaths of tribal elders without the transfer of traditional skills to other members of the family, the migration of people due to social problems, urbanization and modernization, and the influence of modern medicine and exotic cultures. Therefore, the general research objective of this study was to collect, identify, and document medicinal plants and to collate the associated indigenous knowledge of the Sidama people with regard to how they treat various human and livestock ailments in the Hawassa Zuria district of the Sidama zone of Southern Ethiopia. This study was also conducted with the following specific objectives: (1) to measure and compare the indigenous knowledge of the Sidama people among social groups, (2) to discover traditional knowledge gaps and threats to medicinal plants, and (3) to provide baseline data for further phytomedicine and phytochemical studies.

## Material and methods

### Description of the study area

**Fig. 1:**
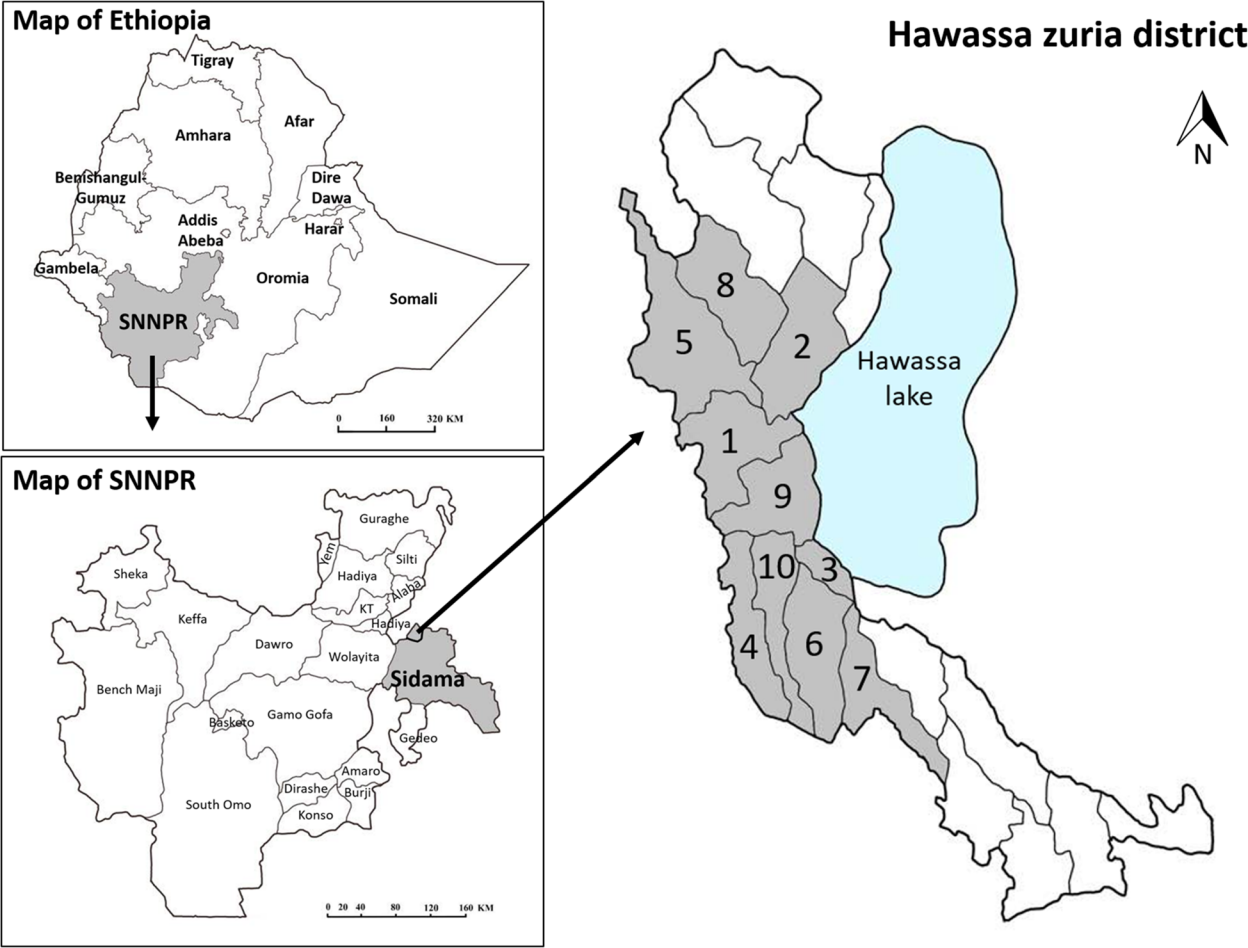
Map of Hawassa Zuria district, Sidama zone, Southern Ethiopia. Study area kebeles: (1) Dore Bafeno, (2) Galo Argiso, (3) Jara Damowa, (4) Jaro Dado, (5) Lebu Korem, (6) Jara Hirnesa, (7) Tenkaka Umbulo, (8) Doyo Otilcho, (9) Jara Gelalcha, (10) Jara Qerara

The Hawassa Zuria district (07° 01′ 54″ to 07° 50′ 36″ N and 38° 15′ 39″ to 38° 25′ 43″ E) is located 290 km from Addis Ababa in the Sidama zone, Southern Nations, Nationalities, and Peoples’ Region (SNNPR) of Ethiopia, bordering Tula town in the east, Lake Hawassa in the north, the Oromia region in the west, and the Boricha district to the south (Fig. 1). This district has a total population of 124,472, of whom 62,774 are men and 61,698 women [7]. The altitudinal range is 1700 m to 1850 m.a.s.l. The annual mean maximum and minimum temperatures are 30 °C and 17 °C, respectively, and the mean annual rainfall is 1015 mm. The size of the district is 22,643 ha and the dry zone accounts for 75% [29] and consists of 23 kebeles (farmers’ associations).

The study was conducted in ten kebeles in the Hawassa Zuria district, SNNPR, from January to February of 2018. The ten kebeles selected were ‘Dore Bafeno,’ ‘Galo Argiso,’ ‘Jara Damowa,’ ‘Jara Dado,’ ‘Tenkaka Unbulo,’ ‘Lebu Korem,’ ‘Jara Qerara,’ ‘Jara Hirnesa,’ ‘Jara Gelalcha,’ and ‘Doyo Otilcho’ (Fig. 1). The criteria for the selection of these study sites were the availability of traditional healers and recommendations from older people and local authorities.

### Sampling informants

In total, 150 informants (118 men and 32 women) were selected among the Sidama people in the Hawassa Zuria district based on recommendations of elders, village administrators, and local guides. The ages of the informants were between 20 to 93 years. Snowball sampling was used and appointments were made prior to visiting the key informants.

### Ethnobotanical data collection

The ethnobotanical study was collected from January to February of 2018. The techniques employed for data collection were group discussions, field observations, guided field walks, and a semi-structured questionnaire (see Additional file 1: Table S1). A semi-structured questionnaire that focused on determining the socio-economic status of the participants was prepared, and both informants and key informants were asked to present their knowledge about the medicinal plants they used to treat different ailments, the plant parts used, the method of preparation for the remedy, and details of the administration method and uses other than medicinal.

General and key informants were interviewed in the Sidama language with the assistance of a native translator.

### Plant specimen collection and identification

Sample specimens of the plants cited for their medicinal use were collected, numbered, pressed, and dried for identification. Preliminary plant identification was performed in the field and results were reconfirmed at the National Herbarium of AAU. Identification of plant specimens was done using the Flora of Ethiopia and Eritrea book and also by comparisons with authenticated specimens with the help of experts at the National Herbarium of Addis Ababa University. Voucher specimens were deposited in the National Herbarium of Addis Ababa University.

### Data analysis

Descriptive statistical methods, in this case percentage and frequency, were used to analyze and summarize the data on medicinal plants as well as their uses and associated knowledge, with MS Excel. According to disease categories in earlier work [30] and with some modification, the ailments were categorized into 14 categories based on the usage reports mentioned by the informants in the study area. The collected data were analyzed through the informant consensus factor and fidelity level [31, 32].

### Quantitative analysis

The informant consensus factor was calculated using the formula ICF = nur-nt/nur-1, where ICF denotes the informant consensus factor, nur is number of use citations, and nt is the number of species used [33]. ICF values range from 0.00 to 1.00. High ICF values are obtained when only one or a few plant species are reported to be used by a high proportion of informants to treat a particular ailment. Low ICF values indicate that informants disagree over which plant to use. High ICF values can thus be used to find particularly important species in searches of bioactive compounds [34]. Fidelity level was used to analyze plant use with the formula FL = Np/N*100, where Np denotes number of informants who reported the use of the plant to treat a particular disease and *N* represents the number of informants who used the plants as a medicine [33].

Jaccard’s coefficient of similarity (JCS) was calculated to evaluate medicinal plant species compositions and degrees of similarity among different areas. Similarity values were calculated between the present study area (Hawassa Zuria district) and other areas in similar agroecological zones which had been studied by other researchers in different parts of Ethiopia. The formula used to calculate the JCS is JCS = *c*/(*a* + *b* + *c*), where JCS is Jaccard’s coefficient of similarity, *a* is the number of species found in habitat A, *b* is the number of species found only in habitat B, and *c* is the number of common species found in habitats A and B [35].

### Preference ranking

Key informants were selected to assess the degree of effectiveness of medicinal plants when used to treat human and livestock diseases following Martin [36]. The medicinal plants believed to be most effective to treat an illness were given the highest value (5), while the least effective received the lowest values (1). The value of each species was summed and the rank for each species was determined based on the total score. This helped to indicate the most effective medicinal plants used by the community to treat diseases.

### Direct matrix ranking

By following Cotton [37], direct matrix ranking was conducted in order to compare multipurpose medicinal plants commonly reported by informants. Based on the relative benefits obtained from each plant, eight multipurpose plant species were selected and seven use diversities of these plants were listed. Three key informants were chosen to assign use values for each attribute (5 = best, 4 = very good, 3 = good, 2 = less, 1 = least used). The use categories include food, fodder, house construction, farming utensils, material cleaning, cultural value, and firewood. Based on data obtained from the informants, the average use diversity value for each species was determined and the values for each species were finally summed and ranked.

## Results

### Medicinal plants in the Hawassa Zuria district

**Fig. 2:**
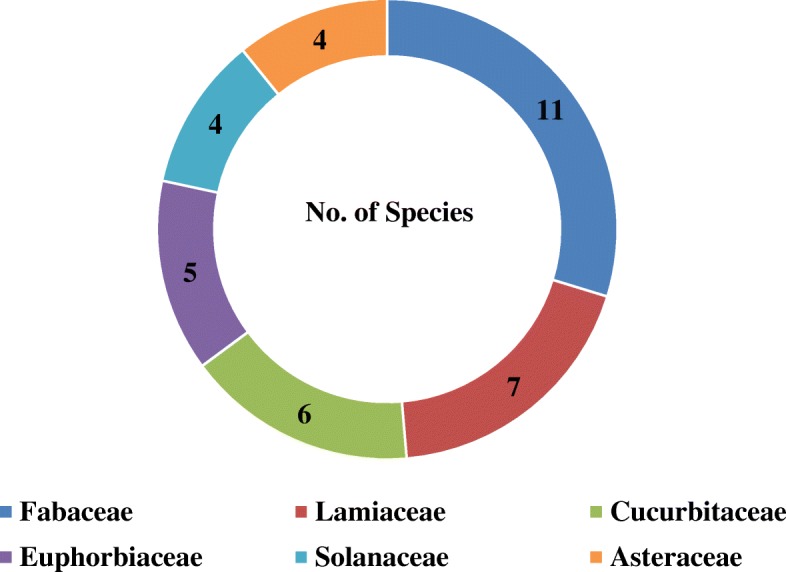
Distribution of medicinal plant species across the different families

A total of 105 medicinal plant species belonging to 95 genera and 52 families were recorded in the study area (Table 1). Fabaceae (11 species) was represented by the highest number of plant species, followed by Lamiaceae (seven species), Cucurbitaceae (six species), Euphorbiaceae (five species), Solanaceae and Asteraceae (four species each) (Fig. 2). Anacardiaceae, Boraginaceae, Capparidiaceae, Malvaceae, Myrtaceae Poaceae, and Rutaceae were represented by three species each, whereas Celastraceae, Meliaceae, Moraceae, Musaceae, Rosaceae, Rubiaceae, and Verbenaceae were represented by two species each. Each of the remaining families was represented by one species (see Additional file 2: Table S2).

**Table 1:** List of medicinal plants used by the Sidama people in the Hawassa Zuria district

No.	Family	Species	Vernacular name	Voucher number	Habit	Plant part used	Methods of preparation	Used to treat	Ailments
1	Acanthaceae	*Acanthus eminens* C. B. Clarke	Amesa buticho (Sd)	BN030	S	L, R	Boiling, chewing, spitting, liquid form	HL	‘Fancho,’ snakebite, wound, menstrual problem
2	Amaryllidaceae	*Allium sativum* L.	Netch shinkurt (Amh)	BN077	H	Bu, Fr, L	Eating, grinding, chewing, spitting	HL	Abdominal pain, malaria, ‘mitch’
3	Amaranthaceae	*Amaranthus caudatus* L*.*	Gerbabo (Sd)	ET053	Cl	R	Chewing, spitting	H	Cancer
4	Anacardiaceae	*Schinus molle* L.	Qundo berbere (Amh)	ET054	T	Ba, L	Chewing, squeezing	H	Toothache, ‘mitch,’ housefly
5	Anacardiaceae	*Mangifera indica* L.	Mango (Amh)	BN092	T	Fr	Eating	H	Disease protector
6	Anacardiaceae	*Searsia natalensis* (Bernh. ex C.Krauss) F.A.Barkley	Dawowesa (Sd)	ET001	T	L	Chewing, grinding, spitting, powdering	HL	Stomachache, snakebite, weight gain
7	Apocynaceae	*Carissa spinarum* L.	Gora (Sd), Agam (Amh)	BN046	Cl	R	Boiling	H	Diarrhea
8	Araceae	*Colocasia esculenta* (L.) Schott	Qolchoma (Sd)	BN068	T	L	Boiling, drinking	L	Placenta delay
9	Asteraceae	*Vernonia filigera* Oliv. & Hiern	Rejicho (Sd)	ET012	S	L	Grinding	H	Leg pain
10	Asteraceae	*Laggera crispata* (Vahl) Hepper & J.R.I.Wood	Amesa maeshana (Sd)	ET026	H	L	Chewing, spitting	HL	Ear disease
11	Asteraceae	*Echinops kebericho* Mesfin	Bursa (Sd), Kebricho (Amh)	BN066	H	R	Smoke form	H	Headache
12	Asteraceae	*Vernonia schimperi* DC.	Hecho (Sd), Gerawa (Amh)	BN076	H	Sh, Ba, L, R, St, Fr	Grinding, powdering, chewing, spitting, sprinkling, smoking	HL	Abdominal pain, wound, headache, back pain, ‘gadanesa’
13	Balanitaceae	*Balanites aegyptiaca* (L.) Delile	Godicho (Sd)	ET043	T	Fr	Chewing, liquid form, grinding	HL	Stomachache, diarrhea, headache
14	Boraginaceae	*Cynoglossum coeruleum* Hochst. ex. A.DC.	Hifaticho (Sd), Chigogit (Amh)	ET007	H	R	Chewing, spitting	H	Cancer
15	Boraginaceae	*Ehretia cymosa* Thonn.	Gidincho (Sd)	ET021	S	L, Ba	Chewing, grinding, boiling, squeezing, spitting	HL	Cancer, toothache, wound
16	Boraginaceae	*Cordia africana* Lam.	Wanza (Amh)	ET033	T	Ba	Chewing	H	Stomachache
17	Brassicaceae	*Brassica carinata* A. Braun	Ye habesha gomen (Amh)	BN089	H	L, Fr, St	Grinding, liquid form, rub	L	Eye wound, swelling of stomach, abdominal pain
18	Capparidaceae	*Capparis tomentosa* Lam.	Gaho (Sd), Gumore (Amh)	BN067	S	R, Fr	Grinding, chewing	HL	Abdominal pain, cancer, tonsillitis
19	Capparidiaceae	*Maerua aethiopica* (Fenzl) Oliv.	Qontir firae (Amh)	BN091	S	Fr	Chewing	H	Cancer
20	Capparidiaceae	*Boscia angustifolia* A. Rich.	Shisha (Sd)	BN101	S	L	Boiling	H	Swelling, butter sweetener
21	Caricaceae	*Carica papaya* L.	Papaya (Amh)	ET082	T	Fr, L, Lx, St, R	Eating, liquid form, squeezing, grinding,	H	Gastritis, malaria, tapeworm
22	Celastraceae	*Gymnosporia senegalensis* (Lam.) Loes.	Chucho (Sd)	ET032	S	L	Chewing, spitting	L	Swelling
23	Celastraceae	*Catha edulis* (Vahl) Endl.	Chat (Amh)	ET050	S	L	Eating, boiling, chewing, spitting, mixed with water	H	Gastritis, gonorrhea, toothache, evil eye
24	Commelinaceae	*Commelina africana* L.	Lalonxe (Sd)	ET037	H	L, St	Rubbing, cutting grinding, liquid form	HL	Skin disease, chirt, quaqucha
25	Convolvulaceae	*Ipomoea batatas* L. Lam.	Maxaxurisha (Sd)	BN064	H	R	Chewing, spitting	H	Wound
26	Crassulaceae	*Kalanchoe petitiana* A. Rich.	Hanshulule (Sd)	ET016	H	L, Bu, R, Fr	Chewing, eating, grinding, squeezing	HL	Dingetegna,gastritis, eye pain
27	Cucurbitaceae	*Peponium vogelii* (Hook.f) Engl.	Surupa (Sd)	ET014	H	L, Fr	Grinding, eating, liquid form, boiling	HL	Malaria, wounds, stomachache
28	Cucurbitaceae	*Cucurbita pepo* L.	Balaqa (Sd)	ET052	H	L	Grinding	H	Swelling
29	Cucurbitaceae	*Cucumis dipsaceus* Ehrenb. ex Spach	Basu baqula (Sd)	ET055	H	Fr, R, Ba, L	Grinding, chewing, hold on teeth	HL	Cough, cancer, black leg
30	Cucurbitaceae	*Lagenaria siceraria* (Molina) Standl.	Buqe (Sd), Kel (Amh)	ET058	H	L	Grinding	H	Liver problem
31	Cucurbitaceae	*Momordica foetida* Schumach.	Herasae (Sd)	BN102	H	L	Liquid form, grinding	HL	Livestock disease, gastritis
32	Cucurbitaceae	*Momordica boivinii* Baill.	Kirae (Sd)	BN103	Cl	L, Fr	Grinding, eating, boiling, squeezing	HL	Stomachache, evil eye, weight gain
33	Cupressaceae	*Juniperus procera* Hochst. ex Endl.	Honicho (Sd)	BN072	T	L	Grinding	L	Diarrhea
34	Dioscoreaceae	*Dioscorea bulbifera* L.	Harae (Sd)	BN093	Cl	R, L	Grinding, chewing, spitting squeezing	HL	Gadanesa, evil eye, ‘fancho’
35	Euphorbiaceae	*Croton macrostachyus* Hochst. ex Delile	Besana (Amh): Masincho (Sd)	ET022	T	Sh, Fr, L	Grinding, liquid form, chewing, boiling	HL	Tuberculosis, gastritis, goiter
36	Euphorbiaceae	*Euphorbia abyssinica* J.F.Gmel.	Qulqual (Amh)	ET004	T	R, L, Lx, Fl, Fr, Ba	Chewing, liquid form, rubbing	HL	Swelling, gastritis, malaria, headache
37	Euphorbiaceae	*Tragia brevipes* Pax	Sonicho (Sd)	ET029	H	L, Fr, Fl	Grinding, eating	L	Evil eye, cancer, anthrax, diarrhea
38	Euphorbiaceae	*Ricinus communis* L.	Gulo (Amh)	ET018	S	R, Fr, L	Grinding, chewing, spitting	HL	Coughing, constipation, swelling
39	Euphorbiaceae	*Euphorbia tirucalli* L*.*	Qinchib, Maxo (Amh)	BN100	S	Lx	Cutting and drop the milk	H	‘Kintarot’
40	Fabaceae	*Senna didymobotrya* (Fresen.) H.S.Irwin & Barneby	Chebicha (Sd)	ET017	S	L	Grinding, squeezing, liquid form, chewing	HL	‘Mitch,’ wound, eye disease, diarrhea
41	Fabaceae	*Millettia ferruginea* (Hochst.) Baker	Galachach (Sd)	ET047	T	Ba	Grinding, mix with water, chewing	H	‘Woranto,’ toothache
42	Fabaceae	*Albizia gummifera* (J.F.Gmel.) C.A.Sm.	Maticho (Sd), Sensel (Amh)	ET044	T	L, Fr, Ba, R, St	Grinding, boiling	HL	Swelling of stomach, evil eye
43	Fabaceae	*Vigna* sp*.*	Mae chorqaye (Sd)	ET040	Cl	L, Fr	Eating, grinding squeezing	HL	Gastritis, cancer, headache
44	Fabaceae	*Acacia etbaica* Schweinf.	Xedecha (Sd)	ET041	T	L, Ba	Powdering, grinding, chewing, spitting	L	Wound, cancer, swelling
45	Fabaceae	*Acacia abyssinica* Benth*.*	Doma Chucho (Sd)	ET036	T	L	Rubbing, spitting	L	‘Balaamo’
46	Fabaceae	*Calpurnia aurea* (Aiton) Benth.	Chekata, Chikea (Sd)	BN062	S	L, R, Sh	Chewing, rubbing, powdering, grinding	HL	Gastritis, stomachache, toothache,
47	Fabaceae	*Acacia albida* Delile	Bura (Sd)	BN065	T	St	Chewing, brushing	H	Toothache
48	Fabaceae	*Senna septemtrionalis* (Viv.) H.S. Irwin & Barneby.	Hamashaka (Sd)	BN069	H	L	Rubbing	H	Snakebite
49	Fabaceae	*Trigonella foenum*-*graecum* L.	Abish (Sd)	BN079	H	St, Fr, L, R	Powdering, grinding, boiling, liquid form	H	Amoeba, weight gain, stomachache
50	Fabaceae	*Acacia seyal* Delile	Girar (Amh), Wacho (Sd)	BN090	T	L	Chewing, spitting	H	Cancer
51	Lamiaceae	*Premna schimperi* Engl.	Udo (Sd)	BN063	S	L, R, St	Grinding, chewing, rubbing, boiling	HL	Toothache, coughing, stomachache
52	Lamiaceae	*Satureja punctata* (Benth.) R.Br. ex Briq.	Amesa (Sd)	BN096	H	L	Boiling	H	‘Shifeta’/‘fancho’
53	Lamiaceae	*Salvia nilotica* Juss. ex Jacq*.*	Kot jebesa (Sd)	BN070	H	L,R, Ba	Chewing, spitting, grinding, rubbing	HL	‘Mitch,’ skin disease, toothache
54	Lamiaceae	*Ocimum gratissimum* L.	Damakese (Amh), Angabisha (Sd)	ET015	S	L, R	Grinding, eating, smelling	HL	‘Mitch’, malaria, cancer
55	Lamiaceae	*Plectranthus igniarius* (Schweinf.) Agnew	Tonton (Sd)	ET059	H	L	Grinding, rubbing, squeezing	H	Evil eye, sun problem, wound, weight loss
56	Lauraceae	*Persea americana* Mill.	Avocado (Amh)	BN085	T	Fr	Eating	H	Diabetes, dandruff
57	Lamiaceae	*Rotheca myricoides* (Hochst.) Steane & Mabb.	Madisisa (Sd)	BN025	S	Ba, L, R	Rubbing, grinding, eating, boiling	HL	‘Mitch,’ cancer, toothache
58	Lamiaceae	*Ajuga integrifolia* Buch.-Ham.	Anamuro (Sd)	BN031	H	L	Grinding, drinking	H	Stomachache
59	Linaceae	*Linum usitatissimum* L.	Shelala (Sd), Telba (Amh)	BN083	H	St	Grinding, liquid form	H	Weight gain, asthma, liver disease
60	Malvaceae	*Ceiba pentandra* L. Gaertn.	Ye tit firea (Amh)	ET006	T	Fr	Grinding, hold on teeth	HL	Toothache
61	Malvaceae	*Sida rhombifolia* L.	Chikicho (Sd)	ET027	H	R	Powdering, mix with water	H	Abdominal pain
62	Malvaceae	*Sida ovata* Forssk.	Qirqicha (Sd)	BN104	S	L, R	Liquid form	H	Toothache
63	Meliaceae	*Azadirachta indica* A. Juss.	Mimi (Sd), Neem (Amh)	ET005	T	L, Ba, R	Grinding, chewing, boiling, liquid form	H	Malaria, toothache, weevils, stomachache
64	Meliaceae	*Ekebergia capensis* Sparrm.	Oloncho (Sd)	BN098	T	L, Ba, St	Boiling, grinding	HL	Goiter, tuberculosis, anthrax
65	Melianthaceae	*Bersama abyssinica* Fresen*.*	Teberako (Sd)	ET048	S	Ba, St, Fr, L	Grinding, chewing, spitting, eating, burning	HL	Cough, headache, stomachache
66	Menispermaceae	*Stephania abyssinica* (Quart.-Dill. & A.Rich.) Walp.	Kelala (Sd)	BN105	H	L, R, Fr	Grinding, boiling, liquid form,	HL	‘Gadanesa,’ eye disease, amoeba
67	Moraceae	*Ficus vasta* Forssk.	Wadicho (Sd), Warka (Amh)	ET034	T	Ba	Eating, chewing	HL	Stomachache, bloody urine, toothache
68	Moraceae	*Ficus sur* Forssk.	Odako (Sd), Ye shola zaf (Amh)	ET035	T	Fr	Eating	H	Stomach ache
69	Moringaceae	*Moringa stenopetala* (Baker f.) Cufod.	Haleqo (Sd), Shiferaw (Amh)	ET009	T	L, R, Ba, Fr	Grinding, boiling, hold on teeth, chewing	HL	Amoeba, hypertension, malaria
70	Musaceae	*Ensete ventricosum* (Welw.) Cheesman.	Enset (Amh), Wesse (Sd)	BN075	S	L, R, Bu	Boiling, chewing	HL	Placenta delay, weight gain
71	Musaceae	*Musa accuminata* Colla	Muz (Amh)	BN088	H	L	Liquid form	H	Wound, cancer
72	Myrtaceae	*Psidium guajava* L.	Zeytuna (Amh)	ET010	T	Fr	Eating	H	Eye disease, gastritis, worms, headache
73	Myrtaceae	*Eucalyptus globulus* Labill.	Netch bahir zaf (Amh)	ET023	T	L, Fr, Fl, Sh	Rubbing, smelling, squeezing, chewing, grinding, liquid form	H	Headache, mitch, stomachache, cough, common cold
74	Myrtaceae	*Eucalyptus camaldulensis* Dehnh.	Tikur bahir zaf (Amh)	ET042	T	L	Rubbing, hold on teeth	H	Toothache
75	Oleaceae	*Olea welwitschii* (Knobl.) Gilg & G. Schellenb*.*	Woiera (Amh)	ET019	T	L, R, Sh, St	Squeezing, chewing, smoking, grinding, spitting	HL	Eye disease, headache
76	Oliniaceae	*Olinia rochetiana* A. Juss	Nole (Sd)	BN097	T	L, R, Ba	Grinding, Boiling, Rubbing	HL	Evil eye, cancer, diarrhea
77	Papaveraceae	*Argemone mexicana* L.	Kokole (Sd)	ET008	H	Sh, L, R, St	Liquid form, grinding, powdering, chewing	HL	Wound, headache, malaria,
78	Poaceae	*Hordeum vulgare* L.	Gebis (Amh)	BN086	H	St	Grinding, mix with water	H	Weight gain
79	Poaceae	*Eleusine coracana* (L.) Gaertn.	Dagusa (Amh)	BN087	H	St	Grinding, mix with food	H	Back pain
80	Poaceae	*Triticum dicoccon* (Schrank) Schübl.	Aja (Amh)	BN080	H	St, Fr	Boiling, grinding, roasting	H	Weight gain, bone fractures
81	Podocarpaceae	*Afrocarpus falcatus* (Thunb.) C.N. Page	Dagucho (Sd)	BN056	T	L	Grinding	HL	Malaria, ‘magarto,’ ‘gadanesa’
82	Polygonaceae	*Rumex abyssinicus* Jacq.	Shoshone (Sd), Meq meqo (Amh)	BN073	H	Ba, R, St	Eating, grinding, mix with boiled water,	H	Stomachache, ‘kintarot’
83	Primulaceae	*Maesa lanceolata* Frossk.	Gowacho (Sd)	BN061	S	L, Ba	Grinding, boiling	HL	‘Gadanesa’
84	Phytolaceae	*Phytolacca dodecandra* L’Hér.	Haranjicho (Sd)	ET051	S	L, R, Fr	Grinding, powdering, chewing,	HL	Cancer, ‘gadanesa’, wound
85	Ranunculaceae	*Nigella sativa* L.	Tiqur Azemud (Amh)	BN078	H	Fr, St	Chewing, grinding, powdering, boiling, smelling	H	Stomachache, gastritis, headache,
86	Rhamnaceae	*Rhamnus prinoides* L′Hér.	Gesho (Amh)	ET002	S	Fr, L	Grinding, chewing, rubbing	H	‘Quaqucha’,stomach-ache, gastritis, wound
87	Rosaceae	*Rubus steudneri* Schweinf.	Gore (Sd), Enjory (Amh)	BN074	H	St	Liquid form	H	Child cleaning
88	Rosaceae	*Hagenia abyssinica* (Bruce ex Steud.) J.F.Gmel.	Koso zaf (Amh)	BN081	T	L, Fl, Fr	Grinding	HL	Diarrhea, weight gain, cancer
89	Rubiaceae	*Coffea arabica* L.	Buna (Amh)	ET057	T	L	Boiling, drinking	H	Gastritis, ‘fancho,’ worms
90	Rubiaceae	*Gardenia ternifolia* Schumach. & Thonn.	Gambella (Sd)	BN099	T	L	Boiling, grinding rubbing	L	Evil eye, ‘gadanesa’
91	Rutaceae	*Casimiroa edulis* La Llave.	Kazmir (Amh)	ET039	T	Fr	Eating	H	Gastritis, stomachache
92	Rutaceae	*Ruta chalepensis* L.	Tena adam (Amh)	ET084	H	L, Sh, St	Grinding, liquid form	H	Amoeba, headache, gonorrhea
93	Rutaceae	*Citrus aurantiifolia* (Christm.) Swingle	Lomi (Amh)	BN094	T	Fr	Eating	H	Gastritis, low blood pressure, hypertension
94	Santalaceae	*Osyris quadripartita* Salzm. ex Decne.	Tunto (Sd)	ET045	S	L, R	Squeezing, powdering, grinding, liquid form	HL	Stomachache, cough, swelling
95	Sapindaceae	*Dodonaea viscosa subsp. angustifolia* (L.f.) J.G.West L.f.	Ittancha (Sd)	ET011	S	L	Boiling, grinding, squeezing,	HL	Evil eye, diarrhea, ticks
96	Solanaceae	*Solanum incanum* L.	Borbodicho (Sd) Embuaye (Amh)	ET013	S	Fr, R, St	Grinding, chewing, spitting, rubbing	HL	Cough, stomachache
97	Solanaceae	*Solanum americanum* Mill*.*	Tunayae (Sd)	ET003	S	L, Fr	Grinding, boiling, chewing, eating	HL	Malaria, toothache, snakebite
98	Solanaceae	*Datura stramonium* L.	Banje (Sd)	ET020	H	Fr, L, St	Grinding, rubbing, liquid form, boiling	HL	Toothache, malaria, ‘Dingetegna’
99	Solanaceae	*Nicotiana tabacum* L.	Arado (Sd), Timbaho (Amh)	BN060	H	L	Boiling, chewing, spitting	HL	Toothache, snakebite, headache, tick
100	Unidentified	Unidentified	Gagasa (Sd)	ET024	Un	L	Grinding, drying, burning	H	Leech, cockroach
101	Verbenaceae	*Lantana camara* L.	Ye wof kolo (Amh)	ET038	S	Fr	Grinding, rubbing	H	‘Chirt’
102	Verbenaceae	*Lippia adoensis* Hochst.	Hanasho (Sd), Kosert (Amh)	BN071	S	L	Eating	H	Stomachache
103	Vitaceae	*Ampelocissus bombycina* (Baker) Planch.	Molama (Sd)	ET049	Cl	R	Grinding	H	‘Azurit’ disease
104	Xanthorrhoeaceae	*Aloe adigratana* Reynolds.	Argisa (Sd) Eret (Amh)	ET028	S	L, Ba, R, St	Liquid form, rubbing, grinding	HL	Skin disease, gastritis
105	Zingiberaceae	*Zingiber officinale* Roscoe	Zinjibil (Amh)	BN095	S	L	Chewing, spitting	H	Eye disease

Key: Vernacular name: Amh = Amharic, Sd = Sidamigna; Used to treat: H = human, L = livestock, HL = human and livestock; Habit: S = shrub, T = tree, Cl = climber, H = herb; un = unknown; plant parts used: R = root, L = leaf, St = stem, Bu = bulb, Lx = latex, Fl = flower, Sd = seed, Sh = shoot, Fr = fruit, Ba = bark; plant names are checked based on http://www.theplantlist.org

### Endemic medicinal plant species

Among the documented medicinal plants in the study area, 71% of species were indigenous and 20% were introduced to Ethiopia. Five endemic plant species were recorded, representing 5% of all medicinal plant species in this study (Table 2).

**Table 2:** Endemic plant species found in the study area

No.	Species	Vernacular name	Family	IUCN category
1	*Echinops kebericho* Mesfin	Bursa (Sd)	Asteraceae	VU
2	*Kalanchoe petitiana* A. Rich.	Hanshulule (Sd)	Crassulaceae	LC
3	*Millettia ferruginea* (Hochst.) Baker	Galachach (Sd)	Fabaceae	LC
4	*Lippia adoensis* Hochst.	Hanasho (Sd),	Verbenaceae	LC
5	*Aloe adigratana* Reynolds	Argisa (Sd)	Xanthorrhoeaceae	VU

*Sd* Sidamigna, *VU* vulnerable, *LC* least concern

### Therapeutic indications

**Fig. 3:**
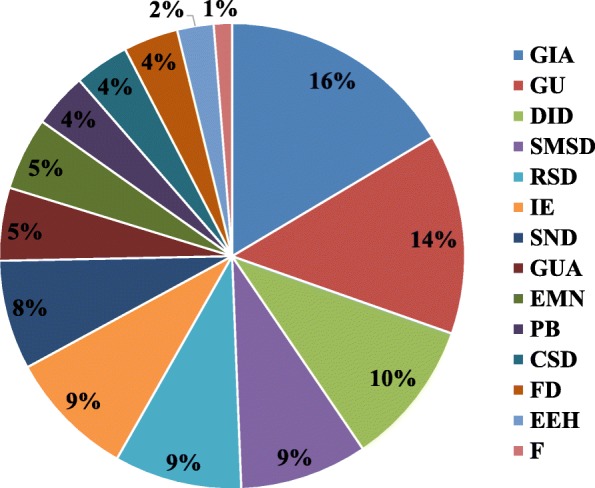
Main therapeutic indications of diseases

In the present study, 48% the reported medicinal plants were used only to treat human diseases, 45% were used for human and livestock ailments, and 8% were used only for livestock diseases. It was also found that 16% of the species were indicated to treat gastrointestinal ailments, followed by general and unspecified diseases at 14%, dermatological infections/diseases at 10%, and skeletomuscular system disorders, respiratory system diseases, and insect and ectoparasites diseases at 9% each (Fig. 3).

### Habits of growth and parts of plants used

**Fig. 4:**
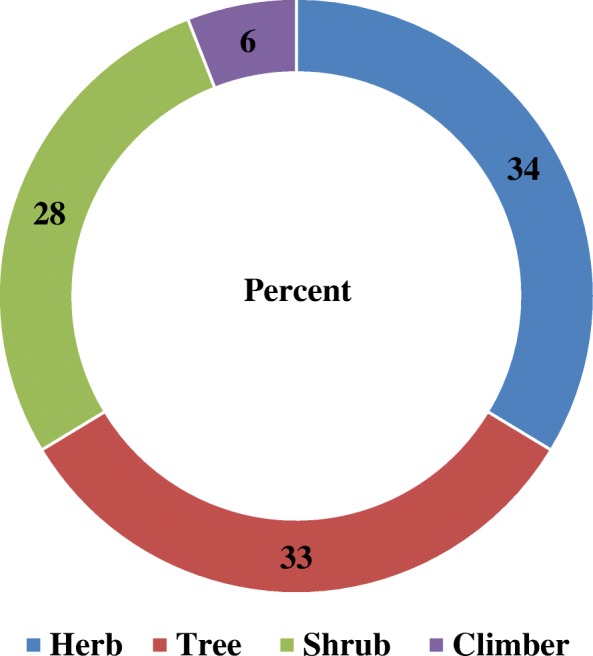
Growth forms of the reported plant species

Medicinal plants were mainly harvested fresh (66%), whereas the remaining (34%) were used in a dry form. The results showed that medicinal plants used to treat human and livestock ailments consisted of 35 herbs (34%), 34 trees (33%), 29 shrubs (28%), and six climbers (6%) species. Thus, the most common form of medicinal plant in the study area was a herb, followed by trees and shrubs (Fig. 4).

**Fig. 5:**
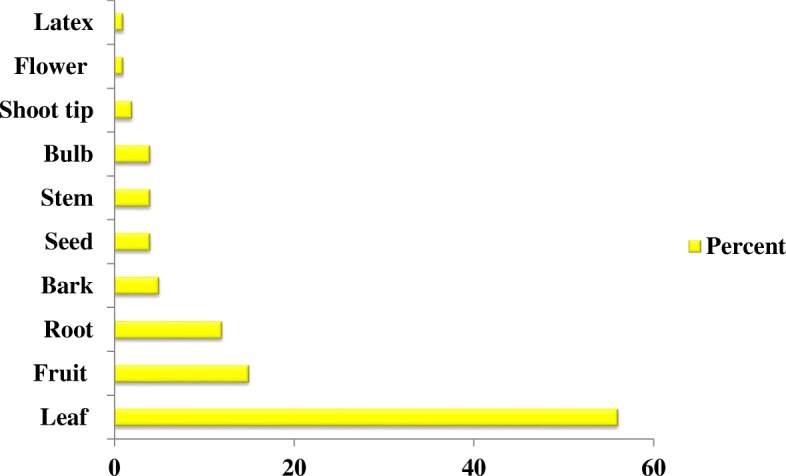
Plant parts used in the study area

The informants of the study area reported that leaves (56%) were the dominant plant part used to prepare remedies, followed by fruits (15%), roots (12%), bark (5%), seeds, stems and bulbs (4%), shoot tips (2%), and flowers and latex (1%) (Fig. 5).

## Methods of preparation and routes of administration

**Fig. 6:**
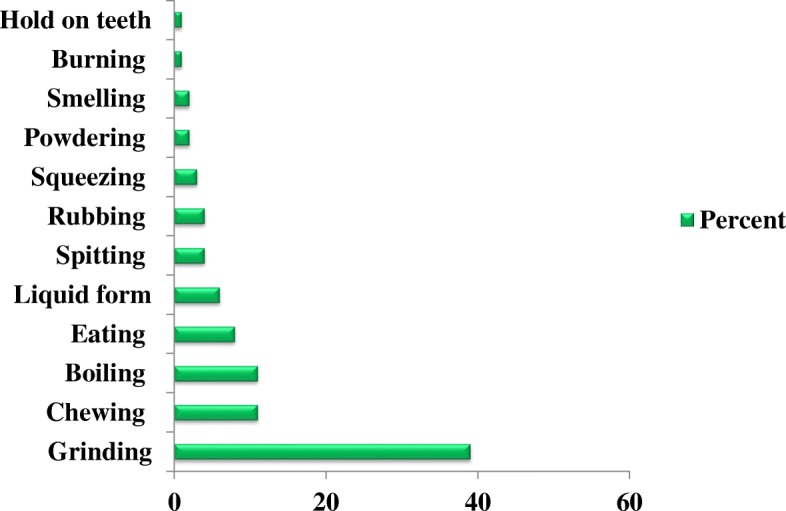
Methods of remedy preparation

The most commonly used methods of remedy preparation were grinding (39%), followed by chewing and boiling (11%), eating (8%), liquid form (6%), spitting and rubbing (4%), squeezing (3%), powdering and smelling (2%), and burning and holding on the teeth (1%) (Fig. 6).

**Fig. 7:**
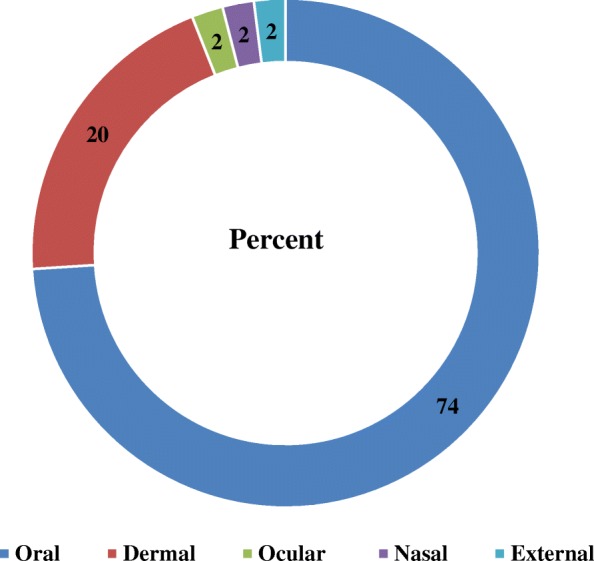
Routes of administration

Medicinal plants were given via different routes of administration, such as oral, dermal, ocular, ear, external, and nasal. The most commonly used route was oral (74%), followed by dermal (20%). The remaining (2%) types were applied through ocular, nasal, and external routes (Fig. 7).

### Medicinal plants used for treatment of human health problems

A total of 50 species belonging to 46 genera and 30 families were recorded to treat human diseases. In the study area, 34 human ailments were identified to be treated by many medicinal plants (see Additional file 3: Table S3.). A single plant can treat a number of human ailments, and a single ailment can be treated by a number of plants. For instance, stomachache is a major disease and can be treated by 41 medicinal plants (Table 3).

**Table 3:** Human ailments that can be treated by medicinal plants

No.	Human ailments	No. of medicinal plants
1	Stomachache	41
2	Headache	28
3	Malaria	18
4	Gastritis	15
5	‘Mitch’	14
6	Amoeba	12
7	Goiter	12
8	Tuberculosis	11
9	Gonorrhea	11
10	Urine problems	10
11	‘Fancho’/‘shifeta’	10
12	Liver disease	10
13	‘Kintarot’	9
14	‘Dingetegna’/Sudden illness	9
15	Hypertension	9
16	Toothache	7
17	Skin disease	6
	Others	70
	Total	276

### Medicinal plants used to treat livestock aliments

A total of eight species belonging to seven genera and seven families were recorded as being used to treat livestock diseases. In this study, livestock diseases are treated with fewer plants compared to those used to treat human diseases. A total of 13 livestock diseases were treated by eight species of plants (Table 4). The most common diseases which affect animals in the study area were wounds, ticks, placenta delay, and swelling.

**Table 4:** Livestock ailments that can be treated by medicinal plants

No.	Livestock ailments	No. of medicinal plants
1	Wounds	6
2	Ticks	5
3	Swelling	3
4	Anthrax	2
5	Evil eye	2
6	‘Gadanesa’	2
7	Abdominal pain	2
8	Bloody urine and shivering	2
9	‘Lelit wof besheta’	2
10	Black leg	1
11	Rabies	1
12	Leech	2
13	Placenta delay	1
	Total	31

### Medicinal plants used to treat both human and livestock health ailments

A total of 47 species belonging to 46 genera and 32 families were recorded as being used to treat both human and livestock diseases. In the Hawassa Zuria district, 22 types of human and livestock ailments were recorded (Table 5) and 47 medicinal plants were identified to treat both human and livestock ailments.

**Table 5:** Human and livestock ailments that can be treated by medicinal plants

No.	Human and livestock ailments	No. of medicinal plants
1	Cancer	19
2	Headache	15
3	Coughing and sneezing	14
4	Diarrhea	14
5	Wound	13
6	Eye pain	13
7	Weight gain	13
8	Toothache	12
9	‘Dingetegna’/Sudden illness	11
10	Worms	10
11	Swelling	10
	Others	62
	Total	207

### Sociodemographic characteristics of the respondents

One hundred fifty informants participated in the ethnobotanical survey of the Hawassa Zuria district. Of these, 118 (78.6%) were men, 32 (21.4%) were women, and 30 were key informants. The majority of respondents were more than 50 years old (42.4%) and 56 informants ranged in age between 36 and 50 years (37.4%). Thirty informants were between 20 and 35 years old (20%). The majority of informants had attended elementary school (48%) (Table 6). Seventy-six percent were farmers and 83% were followers of the protestant religion (see Additional file 4: Table S4).

**Table 6:** Demographic characteristics of the informants

Gender	Count	%	Age	Count	%	Educational status	Count	%
Male	118	78.6	Young (20–35)	30	20	Illiterate	59	39.4
Female	32	21.4	Adult (36–50)	56	37.4	Basic	4	2.6
			Elder (> 50)	64	42.4	Elementary (1–8th)	72	48
						Secondary (9–12th)	12	8
						Tertiary (Tech.)	3	2

### Medicinal plant knowledge with respect to gender, age, and education

Significantly more (*p* < 0.05) medicinal plants were known by men than by women, by informants in the age group above 50 years compared to those aged between 20 and 35 years and between 36 and 50 years, among literate informants who attended elementary school than by illiterate informants (Table 7).

**Table 7:** Comparison of the number of medicinal plants use report by different informant groups

Parameter	Informant group	*N*	Mean ± SD	*p* value
Gender *	Women	117	11.7 ± 8.83	0.000024
	Men	406	40.6 ± 13.6	
Age *	Young (20–35)	106	10.6 ± 8.77	0.039038
	Adult (36–50)	189	18.9 ± 11.9	
	Older (> 50 years)	228	22.8 ± 15.3	
Education *	Illiterate	205	20.5 ± 12.5	0.004907
	Basic	16	1.6 ± 2.83	
	Elementary	256	25.6 ± 11.2	
	Secondary	35	3.5 ± 3.37	
	Tertiary	11	1.1 ± 1.79	

*Significant difference (*p* < 0.05) between the averages of paired categories

### Jaccard’s coefficient of similarity

The highest Jaccard’s coefficient of similarity in the composition of medicinal plants was found between the study area and Wondo Genet district (15.5%), whereas the degree of similarity was lower with Mana Angetu (8.96%) (Table 8). Possible reasons for the similarity and differences between the study area and other areas may be the agroecological climatic conditions in the region.

**Table 8:** Comparison of species found in the study area with those in other study areas

Study areas	Species no. (*a* or *b*)	Common species (*c*)	Jaccard’s coefficient (sj)	% similarity	References
Hawassa Zuria district	105	–	–	–	Study area
Amaro district	56	22	0.12	12.0	[12]
Wondo Genet district	85	35	0.15	15.5	[27]
Wonago district	155	26	0.09	9.09	[3]
Mana Angetu	230	33	0.08	8.96	[49]

*a* is the number of species which is found in habitat A, *b* is the number of species found only in habitat B, and *c* is the number of common species found in habitats A and B

### Informant consensus factor

The diseases in the study area were grouped into 14 disease categories based on the usage reports by the informants and the resemblance to the disease category. A total of 61 diseases treated by 105 plant species were documented in the study area. Among the disease categories, the categories with the highest informant consensus factor (ICF) values were insects and ectoparasites (0.95), followed by fever (0.91) and sensory neuron diseases (0.86) (Table 9). The medicinal plants that had higher ICF values were presumed to be more common and effective when used to treat a certain disease. However, fetal disease and the pregnancy-related category had a lower ICF value (0.59). Lower ICF values indicated that the informants disagreed on the taxa to be used as a treatment within the disease category. The highest plant use citation was found for gastrointestinal issues (418), followed by sensory neuron diseases (297) and then general and unspecified diseases (241).

**Table 9:** Informant consensus factors for categorized diseases

No.	Category name and abbreviations	Reported diseases	No. of usage report	No. of taxa	ICF
1	Gastrointestinal ailments (GIA)	Diarrhea, abdominal pain, swelling of stomach, stomachache, tape worms, amoeba, cholera, gastritis, vomiting, constipation, liver disease, toothache, indigestion	418	101	0.76
2	Dermatological infections/diseases (DID)	Wound, chirt, quaqucha, ‘bugunji,’ itching, skin disease, bijajo (hand wound), dandruff	132	28	0.79
3	Skeletomuscular system disorders (SMSD)	Leg pain, leg swelling, back pain, bone fracture, anthrax, swelling, black leg	67	18	0.74
4	Respiratory systems diseases (RSD)	Tuberculosis, tonsillitis, coughing, sneezing, common cold, cold, and asthma	81	27	0.68
5	Genito-urinary ailments (GUA)	Gonorrhea (STD), ‘kintarot,’ urine problem, kidney disease	69	22	0.69
6	Poisonous bites (PB)	Rabies, Snake bite and Spider poisoning	25	8	0.71
7	Cardiovascular system diseases (CSD)	Hypertension, heart disease, low blood pressure	20	7	0.68
8	Endocrine/metabolic/nutritional (EMN)	Diabetes, weight loss, goiter, weight gain	124	30	0.76
9	Sensory neuron disease (SND)	Cancer, tetanus, headache, brain pain, ‘azurit,’ and typhoid	297	41	0.86
10	Fetal disease pregnancy related (FD)	Abortion, fontanelle closure and placenta delay	18	8	0.59
11	Ear and Eye health (EEH)	Ear disease and eye pain	73	14	0.82
12	Fever (F)	Malaria	79	8	0.91
13	General and unspecified (GU)	Mitch, dingetegna (Wugat, Kurtimat), evil eye, sun problem, Lelit wof disease, Fancho/Shifeta, woranto, kuwashakor, balaamo, magarto, gadanesa	241	61	0.75
14	Insects and ectoparasites (IE)	Cockroach, weevils, honeybee, house fly, corn worm, ticks and leech	164	8	0.95

### Fidelity level

*Eucalyptus globules* (100%) and *Ensete ventricosum* (87.27%) were the two plant species with the highest fidelity levels. These were in the gastrointestinal ailment and fetal disease pregnancy-related categories, respectively, and were followed by *Moringa stenopetala* (81.69%) and *Catha edulis* (74%) correspondingly within the cardiovascular system disease and genito-urinary ailments categories. A higher fidelity level (FL) can imply that a particular plant purpose is preferred if informants mentioned it often. In contrast, the lowest fidelity level value was assigned to *Dodonaea viscosa* subsp*. angustifolia* (29.17%), followed by *Croton macrostachyus* (31.18%) from insects and ectoparasites and the skeletomuscular system disorders category, respectively. A lower fidelity level implies that a particular plant purpose is not preferred (Table 10).

**Table 10:** Relative healing potential levels of 14 cited medicinal plants used against human and livestock ailments

No.	Frequently used species	Particular disease	IP	IU	FL (%)
1	*Eucalyptus globulus*	Stomachache	30	30	100
2	*Rhamnus prinoides*	Skin disease	9	14	64
3	*Croton macrostachyus*	Bone fracture	29	93	31.18
4	*Aloe adigratana*	Coughing	28	44	63.64
5	*Catha edulis*	Kintarot	17	23	74
6	*Moringa stenopetala*	Hypertension	89	109	81.69
7	*Cucurbita pepo*	Weight gain	2	4	50
8	*Cucumis dipsaceus*	Cancer	68	108	62.96
9	*Ensete ventricosum*	Placenta delay	48	55	87.27
10	*Peponium vogelii*	Malaria	28	73	38.36
11	*Vernonia schimperi*	Mitch	25	62	40.32
12	*Dodonaea viscosa subsp. angustifolia*	Ticks	14	48	29.17
13	*Acacia etbaica*	Wounds	10	16	62.5
14	*Ricinus communis*	Cancer	3	6	50
15	*Rotheca myricoides*	Headache	43	95	45.26
16	*Nicotiana tabacum*	Toothache	49	88	55.68

### Preference ranking

Ten key informants were asked to compare seven medicinal plants based on their knowledge of medicinal plants for the treatment of stomach problems by assigning a score of five for the most effective medicinal plants and one for the least effective medicinal plants. *Eucalyptus globulus* Labill. was ranked as the most preferable medicinal plant for stomachache, followed by *Nigella sativa* L. (Table 11).

**Table 11:** Preference ranking of medicinal plants used to treat stomachache in humans

Species	Informants										Total score	Rank
	I1	I2	I3	I4	I5	I6	I7	I8	I9	I10		
*Ruta chalepensis*	4	3	4	5	4	5	2	3	2	3	35	5
*Casimiroa edulis*	3	4	5	4	5	5	5	5	4	4	44	3
*Eucalyptus globulus*	5	5	5	5	5	5	5	5	5	5	50	1
*Azadirachta indica*	2	3	2	2	3	4	3	4	3	4	30	6
*Rhamnus prinoides*	4	4	5	3	3	5	5	4	5	5	43	4
*Nigella sativa*	5	4	4	5	5	5	5	5	5	5	48	2
*Sida rhombifolia*	1	2	2	3	3	2	2	1	1	2	19	7

The output of the preference ranking indicated that *Tragia brevipes* Pax., *Ricinus communis* L., and *Acacia etbaica* Schweinf*.* were the most preferable medicinal plants for treating wound diseases in livestock (Table 12).

**Table 12:** Preference ranking of medicinal plants used to treat wounds in livestock

Species	Informants										Total score	Rank
	I1	I2	I3	I4	I5	I6	I7	I8	I9	I10		
*Tragia brevipes*	5	4	4	5	3	4	4	4	5	5	43	1
*Ricinus communis*	3	3	4	4	3	3	4	2	5	4	35	2
*Acacia etbaica*	2	3	4	3	4	3	2	1	4	3	29	3

According to the results, cancer was the most common disease challenging both humans and livestock in the Hawassa Zuria district. The preference ranking results showed that *Cucumis dipsaceus* Ehrenb. ex Spach was the medicinal plant preferred as a treatment for cancer both in human and livestock, followed by *Rotheca myricoides* (Hochst.) Steane & Mabb. and *Euphorbia abyssinica* J.F.Gmel*.* (Table 13).

**Table 13:** Preference ranking of medicinal plants used to treat cancer in both humans and livestock

Species	Informants										Total score	Rank
	I1	I2	I3	I4	I5	I6	I7	I8	I9	I10		
*Olinia rochetiana*	5	3	3	4	5	4	4	3	4	4	39	4
*Euphorbia abyssinica*	4	4	5	5	5	4	3	4	4	4	42	3
*Rotheca myricoides*	5	5	4	3	5	5	5	5	5	5	47	2
*Cucumis dipsaceus*	5	5	5	5	5	5	5	5	5	5	50	1
*Dodonaea viscose subsp. angustifolia*	3	4	3	5	4	3	3	4	3	4	36	6
*Vernonia schimperi*	3	3	2	4	3	2	3	2	3	2	27	7
*Bersama abyssinica*	5	4	3	3	3	4	3	5	4	3	37	5

### Other uses of medicinal plants in the study area

**Fig. 8:**
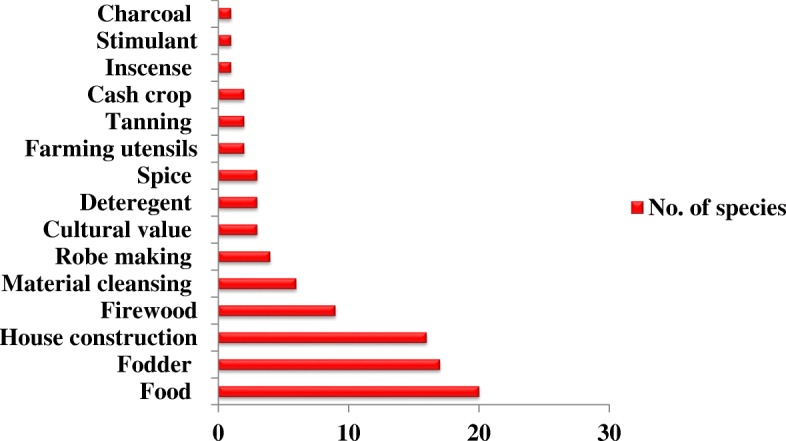
Use categories and number of plant species

The majority of respondents reported that the medicinal plants can also be used as food, fodder, and as house construction materials in the study area (see Additional file 5: Table S5). In this case, 33% of the medicinal plant species were reported to be used for food, the highest rate, followed by fodder (28%), and house construction materials (26%) (Fig. 8).

### Direct matrix ranking

Key informants evaluated the functionality of multifunctional medicinal plants to the local people and indicated their scores for each medicinal plant (on a scale of 1 to 5). Eight medicinal plants were selected to be evaluated in seven usage categories. The output of the direct matrix analysis found *Ensete ventricosum* to be the preferred medicinal plant used for various purposes by the local people, followed by *Olea welwitschii* (Knobl.) Gilg & G. Schellenb., and *Dodonaea viscose* subsp. *angustifolia* (Table 14).

**Table 14:** Direct matrix ranking of medicinal plants by informants (I1--I3) based on usage category values

Plant species	Food			Cultural value			Farming utensils			House construction			Firewood			Fodder			Material cleaning			Total	Rank
	I1	I2	I3	I1	I2	I3	I1	I2	I3	I1	I2	I3	I1	I2	I3	I1	I2	I3	I1	I2	I3		
*Tragia brevipes*	5	4	3	1	2	2	3	5	4	1	2	2	1	2	3	2	1	3	2	3	2	53	7th
*Nicotiana tabacum*	1	3	2	3	4	3	1	1	1	1	3	3	2	3	3	1	4	3	1	2	3	48	8th
*Dodonaea viscose* subsp. *angustifolia*	1	2	2	2	2	1	3	5	4	5	5	5	5	4	5	5	1	4	2	2	1	66	3rd
*Albizia gummifera*	2	3	3	3	3	1	1	2	3	5	4	5	5	5	4	2	1	3	2	3	3	63	5th
*Ensete ventricosum*	5	4	5	4	4	2	3	1	2	5	4	4	4	3	4	5	4	4	3	1	2	73	1st
*Ehretia cymosa*	1	2	3	5	4	1	5	3	3	3	3	3	3	3	3	1	1	2	3	2	2	56	6th
*Acacia etbaica*	1	3	2	4	2	1	5	1	4	5	5	4	5	4	3	2	2	3	2	3	3	64	4th
*Olea welwitschii*	1	2	3	4	4	5	3	1	3	5	4	5	4	4	4	2	3	3	4	4	4	72	2nd
Total		73			80			69			93			82			62			60			
Rank		4th			3rd			5th			1st			2nd			6th			7th			

### Threats to medicinal plants

**Fig. 9:**
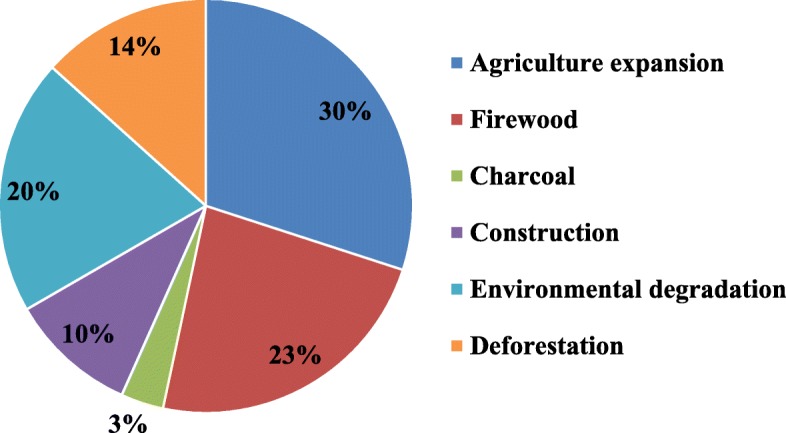
Threats to medicinal plants in the study area

Numerous factors are considered as threats to the medicinal plants in the study area. According to the responses from the key informants, the major threat to medicinal plants was agricultural expansion (30%) followed by firewood collection (23%) and environmental degradation (20%) (Fig. 9).

## Discussion

In this study, 105 medicinal plant species were identified for the treatment of human and livestock ailments distributed across 52 families and 95 genera. From the 52 plant families, Fabaceae (21%) was the major contributing species, followed by Lamiaceae (13%) and then Cucurbitaceae (12%). Similarly, various studies in Ethiopia [25, 38–40] have reported Fabaceae as the most dominant medicinal plant family. In contrast, other studies found that Lamiaceae [18] and Euphorbiaceae [41] were dominant over others.

Among the documented medicinal plants, five endemic medicinal plant species were recorded. Identification of endemic species and current statuses is vital for the conservation of medicinal plants. The medicinal plants in the study area had diverse growth forms, in this case herbs (34%), trees (33%), shrubs (28%), and climbers (6%) were the dominant medicinal plants. Similarly, Lulekal et al. [38] reported that most medicinal plants were herbs in Ankober. Mesfin et al. [3] and Giday [9] also reported that the dominant medicinal plants were herbs. In contrast, the majority of medicinal plants were shrubs in the Wonago district [3]. Regassa [25] in Hawassa city indicated that the majority of the collected medicinal plants there were trees, followed by shrubs.

The results here showed that the local people of the Hawassa Zuria district use different parts of a medicinal plant to prepare remedies. Leaves were the most widely used part, making a contribution to the conservation of plants rather than harvesting the root part and/or whole plant. In the same way, Berhane [20] reported leaves as the predominant plant part used by the Maale and Ari ethnic communities. Ketema [42] also noted leaves were the most commonly used plant part in a study focusing on South Omo. The majority of medicinal plants were harvested for their leaves by the Sheko as well [9]. However, the most frequently used plant parts were roots in the Hadiya zone [43] and in Benna Tsemaye [13].

The local people in the Hawassa Zuria district use different remedy preparation methods depending on the type of disease to be treated. Cutting, tying, spraying, roasting, soaking, hanging, brushing, squeezing, powdering, spitting, and holding on the teeth were some of the preparation methods used to treat human and livestock diseases. The most commonly used method of remedy preparation was grinding (39%), followed by chewing and boiling (11%). Elsewhere in Ethiopia, similar findings were reported [13, 44] with regard to the most commonly used methods of remedy preparation. These were crushing, followed by chewing, boiling, eating, and in liquid form.

The prepared remedies were administered in different ways. Some of the routes of administrations were via the oral, dermal, and ocular routes and via the ear, externally, and through the nasal passage. The majority of the remedies were administered done so orally (74%) followed by the dermal (20%) and ocular (2%) routes. Similar findings were also documented in different parts of Ethiopia. The majority of medicinal plants were administered orally in Endrta [45]. Assegid [13] reported that the majority of plants were used in oral applications in the Tsemay district. Sintayehu [27] found that prepared remedies were widely administered orally in Wondo Genet.

In the Hawassa Zuria district, 34 human, 13 livestock, and 22 ailments of both humans and livestock were recorded. This indicated that the people of the district suffered from many ailments as compared to those in other areas, such as in the Wonago district [3] and the Bench district [9]. Moreover, a single human ailment was found to be treatable by several medicinal plants. This is in agreement with the findings of earlier studies [27, 45] that found single ailments to be treated by several medicinal plants. The present study found that 16% of medicinal plants were used to treat gastrointestinal ailments, with slightly lower rates for general and unspecified diseases (14%). Similarly, among the Sheko, 16.9% of medicinal plants were used to treat gastrointestinal complaints [9]. The highest proportions of Meinit and Dek Island medicinal plants were used to treat gastrointestinal complaints [46, 47].

The present study indicated that men, older, and literate people who attend elementary school have more medicinal plant knowledge as compared to women, younger, and illiterate people. The reason for the high traditional knowledge of men and older people could be due to the influence of modernization and a lack of interest among the younger generations. Similar findings also indicated that there are significance differences in traditional knowledge among different communities in Ethiopia [40, 48]. Unlike other studies, the current study indicated that literate people who attend elementary school reported a higher number of medicinal plants as compared to those who are illiterate.

Jaccard’s coefficient of similarity indicated that there was some similarity in the composition of medicinal plants between the study area and the Wondo Genet district [27], whereas less similarity was found with regard to the Mana Angetu district [49]. This may stem from the agroclimatic conditions of the study area.

The category with the highest ICF values was fever (malaria), followed by insects and ectoparasites diseases. The highest plant use citation was found for gastrointestinal ailments, followed by sensory neuron diseases. Similarly, gastrointestinal disorders and parasite infections were the most commonly treated diseases on the Zegie peninsula [50].

The highest fidelity levels were found for gastrointestinal ailments in this study in all cases. *Eucalyptus globulus* Labill. (100%) scored the highest fidelity level value. Similarly, Reta [25] reported that gonorrhea, wounds, and stomachache had high degrees of ICF and that malaria showed the highest FL in Hawassa city. Teferi [39] indicated that diarrhea and malaria were the most frequently reported diseases among the Benta ethnic group.

The output of the preference ranking indicated that *Eucalyptus globulus*, *Nigella sativa* L., and *Casimiroa edulis* La Llave. were the most commonly preferred medicinal plants as treatment for stomachache in human in the Hawassa Zuria district. Similar findings for South Omo showed that the highest numbers of plant species were reported to treat abdominal or stomach disorders [42]. *Cucumis dipsaceus* Ehrenb. ex Spach, *Rotheca myricoides* (Hochst.) Steane & Mabb., and *Euphorbia abyssinica* J.F.Gmel were ranked as the most preferable medicinal plants to treat cancer in both humans and livestock.

According to the direct matrix ranking results, *Ensete ventricosum* ranked first as the most preferred medicinal plant used for various purposes by the local people. The second and third most preferable medicinal plants were *Olea welwitschii* (Knobl.) Gilg & G. Schellenb. and *Dodonaea viscose* subsp. *angustifolia*, respectively. However, *Prunus africanus* is the most preferred medicinal plant for various use in Hadiya zone [43]. In the present study, *Ensete ventricosum* was used as a type of food, as fodder, in house construction, and for robe making. It also had cultural and spiritual value. *Olea welwitschii* was reportedly used mainly for purposes related to house construction, material cleaning, incense, spiritual value, firewood, and cultural value. However, the overexploitation of medicinal plants for other purposes can affect the availability and conservation of medicinal plants for their primary purpose.

According to the responses from key informants, the main causes of the loss of medicinal plants in the study area were agriculture expansion, firewood collection, environmental degradation, deforestation, construction, and charcoal creation. Other research on threats to medicinal plants in Dale [24], the Benna Tsemay district [13], the Mana Angetu district [49], Wondo Genet [27], Amaro woreda [51], and Wonago woreda [3] indicated findings similar to those here.

Other factors related to the loss of indigenous knowledge about medicinal plants were the secrecy of traditional knowledge practiced by elders in the tribe, a weak transfer system for indigenous knowledge, and the influence of modernization. Similarly, other findings [10, 27, 52–55] reported that there is an aura of top secrecy in the passing of indigenous knowledge within families. Therefore, better conservational awareness by all community members is necessary to retain their own indigenous knowledge and to prevent the extinction of their medicinal plant resources.

### Comparison with previous ethnobotanical studies

*Moringa stenopetala* (Baker f.) Cufod. was the most frequently used plant for amoeba, hypertension, and malaria treatment in the present study and similarly Asnake et al. [23] reported this plant use for malaria in Boricha district. Tamiru and Asalfew [41] reported for inflammation, wound, diabetes, eye disease, and headache treatment in Mirab Badwacho district. Tilahun et al. [56] reported this plant use mainly as vegetable for food. In Wonago district, this plant used for vomiting [3].

*Nicotiana tabacum* L. was the most frequently used plant and reported to be used against Snakebite, toothache, and headache in our study. Similarly, Yigezu et al. [57] and Tamiru and Asalfew [41] mentioned this plant use for snake bite and toothache. This plant in five reports of study in Ethiopia stated its wide spread use in treatment of black leg, tick infestation, wound, leech, diarrhea, and gonorrhea [41, 42, 51, 57, 58].

*Tragia brevipes* Pax was used for evil eye, cancer, anthrax, and diarrhea in the present study, whereas it is used for babesiosis and abdominal pain in other study areas [26, 58]. *Cucumis dipsaceus* was documented for cough, cancer, and black leg treatment in the current study. Asnake et al. [23] and Sintayehu [27] reported this plant use for malaria, intestinal parasite, pneumonia, gonorrhea, and stomach problem.

*Croton macrostachyus* reported in the present study for the treatment of bone fracture, tuberculosis, gastritis, and goiter. Gonfa et al. [24] reported this plant only for tuberculosis purpose in Dale district. Yigezu et al. [57] found out the use of this plant for treatment of bloat in cattle. However, this plant was reported for the treatment of stomachache in Mirab Badwacho district [41], in Dale district [24], and Benna tsemay district [13]. Mirutse [46] and Melesse [44] mentioned the use of this plant for the treatment of snakebite. This plant was reported for the treatment of malaria in Boricha [23] and Amaro district [51]. Other studies reported this plant use for the treatment of wound [24, 51, 58].

*Allium sativum* L. was the most frequently used plant for abdominal pain, malaria, and mitch in the present study. This findings also agreed with the study conducted in Wonago district [3], Mirab Badwacho [41], Kembata [44], and Amaro district [12]. The plant product also has been used for other infections in upper respiratory tract [41]. Miruts et al. [52] and Mesfin et al. [12] reported this plant use for headache in Sheko and Amaro district respectively.

*Aloe adigratana* Reynolds. was a new Aloe species mentioned for the first time in the present study. It was reported in the present study for the treatment of skin disease and gastritis. The previous studies conducted in other area mentioned the use of *Aloe* spp. for cold and malaria treatment in Kembata [44] and Boricha district [23]. *Rotheca myricoides* (Hochst.) Steane & Mabb. also known by the synonym *Clerodendrum myricoides* was reported to be used for mitch, cancer, and toothache treatment in the present study. Similarly, Yibrah [58] reported the use of this plant for teeth pain in Kochore district. Asnake et al. [23] reported the plant to be used against malaria in Boricha district.

*Ensete ventricosum* reported in the present study for placenta delay and weight gain in livestock and human. Unsimilarly, Reta [25], Giday et al. [52], and Andarge et al. [59] reported this plant for diarrhea, bone fracture, and tumor respectively. *Olea welwitschii* was commonly reported to be used for eye disease, headache, and gastritis in this study. Previous studies in Wayu Tuka and Tulu Korma district mentioned this plant for stomachache and gonorrhea disease treatment [60, 61].

*Ricinus communis* reported in the present study for the treatment of coughing, constipation, and swelling. However, Kassa [61] and Amenu [62] mentioned this plant for anthrax treatment in Tulu Korma and Ejaji district. Other studies in south western Ethiopia stated its use in treatment of rabies, sudden illness, blotting, wound, and mastitis [20, 60–63]. *Eucalyptus globulus* was the most frequently used plant and reported to be used for headache, mitch, stomachache, coughing, and common cold in our study. Similarly, it has been reported for common cold and influenza in Wayu Tuka district [60], Gimbi [64], and Dawuro zone [59]. Regassa [25] reported this plant use for malaria, typhoid, Ascarsis, and acute sickness in Hawassa city.

*Nigella sativa* L. was used for stomachache, gastritis, and headache in the present study. Similarly, this plant was reported for stomachache and headache in Hawassa city [25]. Others reported it for asthma, leprosy, and strepto thricosis in Wayu Tuka district [60] and Nekemet [65]. *Acacia etbaica* reported in the present study for the treatment of wound, cancer, and swelling. Similarly, Dinkissa et al. [66] mentioned this plant for wound treatment in Awash National park. However, Eneyew et al. [67] mentioned this plant for snakebite and evileye treatment in Fitche district.

*Euphorbia abyssinica* was reported for swelling, gastritis, malaria, and headache treatment in the current study. Similarly Abera [64] reported it for gastrointestinal disease in Ghimbi district. Ascaris, gonorrhea, warts, rabies, and venereal disease were the diseases mentioned on previous studies [64, 67]. *Dodonaea viscose* subsp. *angustifolia* was the most frequently used plant for evil eye and diarrhea in the present study. This findings disagree with the study conducted in other areas which stated the plant use for bone fracture, herpes, liver ailments wound, and acute sickness [25, 61, 62, 65].

According to the comparison of our findings with others ethnobotanical study in Ethiopia, novel plant uses of some medicinal plants were documented. *Aloe adigratana*, *Tragia brevipes*, *Cucumis dipsaceus*, *Rotheca myricoides*, *Ricinus communis*, and *Dodonaea viscose* subsp. *angustifolia* was completely novel use in our study area and never ever reported in other similar investigations. The pharmacological activity of these plants are novel findings that only known in this area for such medicinal purpose.

## Conclusions

The results of the study revealed that there is high diversity of the medicinal plants in the Hawassa Zuria district. One hundred five medicinal plant species were documented to treat 61 human and livestock ailments. Stomachache, headache, malaria, gastritis, mitch, amoeba, goiter, tuberculosis, gonorrhea, and urine problems were frequently occurring human ailments, whereas wounds, ticks, swelling, anthrax, evil eye, and ‘gadanesa’ were common livestock ailments. This indicates that local people depend on indigenous knowledge to prevent various human and livestock ailments.

In the study area, major knowledge differences were found among different social groups. Male informants had more knowledge than females. Older informants above 50 years of age were more knowledgeable than the young between 20 and 35 years and adult informants between 36 and 50 years. Informants who attended elementary school had more knowledge than those who were illiterate.

The main threats to medicinal plants in the Hawassa Zuria district were deforestation, agricultural expansion, and overexploitation. The medicinal traditional knowledge gap between the older and young generations has also impacted the loss of indigenous knowledge. Therefore, there should be mentoring programs for local people in the study area to conserve their indigenous knowledge resources and prevent the extinction of their medicinal plants.

Conservation of endangered endemic medicinal plants through in vitro and ex vitro propagation should be developed to protect the extinction of medicinal plants. Furthermore, the current documented information on the medicinal plants of the Sidama people can be used as baseline data for future studies of pharmacologically important medicinal plants and for phytochemical investigations.

## Additional files

Additional file 1: **Table S1.** Semi-structured questionnaire, research questions and hypothesis (DOCX 16 kb)

Additional file 2: **Table S2.** List of plant families. (DOCX 18 kb)

Additional file 3: **Table S3.** List of diseases and number of plant species (DOCX 18 kb)

Additional file 4: **Table S4.** Sociodemographic details of the respondents in the Hawassa Zuria district. (DOCX 15 kb)

Additional file 5: **Table S5.** Other uses of medicinal plants in the study area. (DOCX 15 kb)
