# Supplementary material for: Ethnobotanical study of medicinal plants in the Hawassa Zuria District, Sidama zone, Southern Ethiopia
Source: J Ethnobiol Ethnomed. 2019 May 24;15:25. doi: 10.1186/s13002-019-0302-7 (PMC6534827; doi:10.1186/s13002-019-0302-7)
Supplement: Supplementary file 1 — Table S1. Semi-structured questionnaire, research questions and hypothesis (DOCX 16 kb) [file 13002_2019_302_MOESM1_ESM.docx]

**Additional file 1:** **Table S1.** Semi-structured questionnaire, research questions and hypothesis.

**Questionnaire**

Village: _________

Date:

Informant No.:__________

**Participants’ Information:**

Name: Age: Gender: Male/Female

Education status: Marital Status: Single/Married No. of children: _____

Years of residence: ________ Occupation: ____________________ Income: _____________

Religion: __________ Others: ______________________________________________

**Research questions:**

1. What are the major medicinal plants used to treat different aliments?

2. Which part of the plant is used as remedy? (Leaf/Stem/Root/Flower/Bark/Fruit/Seed).

3. What are the methods of preparation of the medicinal plants? (Crushed/pounded/Powdered/Concoction/Decoction/Infusion/mixed with others).

4. Forms of medicinal plants used (Fresh/Dry form).

5. How is the dosage and way of administration of medicinal plants? (Oral/Nasal/Dermal)

6. Place and distance of medicinal plants collection (Home garden/Wild forest).

7. Time of medicinal plants collection (Morning/Afternoon/Night).

8. Side effects and the antidotes while using the medicinal plants.

9. The other uses of medicinal plants (Food/Fodder/Fence/Firewood).

10. Storage and conservation of medicinal plants.

11. Source and transfer of indigenous knowledge (Father/Mother/Friends).

**Hypothesis to be tested:**

- The study area has diverse medicinal plants used for treating different health problems of human and livestock
- Indigenous knowledge on use of medicnal plants varies based on gender, age, and educational level of the local people
- Local ethnobotanical knowledge loss is driven by habitat loss/deforestation, oral based knowledge transfer and modernization
- Traditional practices and knowledge have contribution to conservation of medicinal plants in the study area.
- The medicinal plants of the study area have different plant diversity and types from other study area
