# Supplementary material for: Ethnobotanical study of medicinal plants in the Hawassa Zuria District, Sidama zone, Southern Ethiopia
Source: J Ethnobiol Ethnomed. 2019 May 24;15:25. doi: 10.1186/s13002-019-0302-7 (PMC6534827; doi:10.1186/s13002-019-0302-7)
Supplement: Supplementary file 2 — Table S2. List of plant families. (DOCX 18 kb) [file 13002_2019_302_MOESM2_ESM.docx]

**Additional file 2:** **Table S2.** List of plant families.

| **Family** | **No. of species** | **Percent** | **Family** | **No. of species** | **Percent** |
| --- | --- | --- | --- | --- | --- |
| Fabaceae | 11 | 0.21 | Crassulaceae | 1 | 0.01 |
| Lamiaceae | 7 | 0.13 | Xanthorrhoeaceae | 1 | 0.01 |
| Cucurbitaceae | 6 | 0.11 | Acanthaceae | 1 | 0.01 |
| Euphorbiaceae | 5 | 0.09 | Commeliniaceae | 1 | 0.01 |
| Asteraceae | 4 | 0.07 | Balanitaceae | 1 | 0.01 |
| Solanaceae | 4 | 0.07 | Moraceae | 1 | 0.01 |
| Malvaceae | 3 | 0.05 | Apocynaceae | 1 | 0.01 |
| Myrtaceae | 3 | 0.05 | Vitaceae | 1 | 0.01 |
| Poaceae | 3 | 0.05 | Amaranthaceae | 1 | 0.01 |
| Rutaceae | 3 | 0.05 | Convolvulaceae | 1 | 0.01 |
| Boraginaceae | 3 | 0.05 | Polygonaceae | 1 | 0.01 |
| Anacardiaceae | 3 | 0.05 | Santalaceae | 1 | 0.01 |
| Cappardiaceae | 3 | 0.05 | Melianthaceae | 1 | 0.01 |
| Verbenaceae | 2 | 0.03 | Phytolaceae | 1 | 0.01 |
| Moraceae | 2 | 0.03 | Podocarpaceae | 1 | 0.01 |
| Rubiaceae | 2 | 0.03 | Primulaceae | 1 | 0.01 |
| Celasteraceae | 2 | 0.03 | Ranunnculaceae | 1 | 0.01 |
| Meliaceae | 2 | 0.03 | Caricaceae | 1 | 0.01 |
| Rosaceae | 2 | 0.03 | Linaceae | 1 | 0.01 |
| Musaceae | 2 | 0.03 | Lauraceae | 1 | 0.01 |
| Papaveraceae | 1 | 0.01 | Alliaceae | 1 | 0.01 |
| Araceae | 1 | 0.01 | Oliniaceae | 1 | 0.01 |
| Cupressaceae | 1 | 0.01 | Zingiberaceae | 1 | 0.01 |
| Morigaceae | 1 | 0.01 | Menispermaceae | 1 | 0.01 |
| Rhamnaceae | 1 | 0.01 | Oleaceae | 1 | 0.01 |
| Sapindaceae | 1 | 0.01 | Dioscoreaceae | 1 | 0.01 |
