# Supplementary material for: Ethnobotanical study of medicinal plants in the Hawassa Zuria District, Sidama zone, Southern Ethiopia
Source: J Ethnobiol Ethnomed. 2019 May 24;15:25. doi: 10.1186/s13002-019-0302-7 (PMC6534827; doi:10.1186/s13002-019-0302-7)
Supplement: Supplementary file 3 — Table S3. List of diseases and number of plant species (DOCX 18 kb) [file 13002_2019_302_MOESM3_ESM.docx]

**Additional file 3: Table S3.** List of diseases and number of plant species.

| **No.** | **Human ailments** | **No. of spp** | **Human and livestock ailments** | **No. of spp** |
| --- | --- | --- | --- | --- |
| 1 | Stomachache | 41 | Cancer | 19 |
| 2 | Headache | 28 | Headache | 15 |
| 3 | Malaria | 18 | Coughing and sneezing | 14 |
| 4 | Gastritis | 15 | Diarrhea | 14 |
| 5 | Mitch | 14 | Wound | 13 |
| 6 | Amoeba | 12 | Eye pain | 13 |
| 7 | Goiter | 12 | Weight gain | 13 |
| 8 | Tuberculosis | 11 | Toothache | 12 |
| 9 | Gonorrhea | 11 | Dingetegna | 11 |
| 10 | Urine problems | 10 | Worms | 10 |
| 11 | Fancho/shefeta | 10 | Swelling | 10 |
| 12 | Liver disease | 10 | Evil eye | 9 |
| 13 | Kintarot | 9 | Weight loss | 9 |
| 14 | Dingetegna | 9 | Snake bite | 8 |
| 15 | Hypertension | 9 | Woranto | 6 |
| 16 | Tooth ache | 7 | Sun problem | 6 |
| 17 | Skin disease | 6 | Bone fracture | 6 |
| 18 | Common cold | 6 | Urine problem | 5 |
| 18 | Typhoid | 5 | Skin disease | 5 |
| 19 | Kuwashakore | 5 | Leg pain | 4 |
| 20 | Backpain | 5 | Ear disease | 3 |
| 21 | Fontanelle closure | 2 | Rabies | 2 |
| 22 | Cholera | 2 | **Livestock disease** | **No. of spp** |
| 23 | Tetanus | 2 | Wound | 6 |
| 24 | Tonsillitis | 2 | Ticks | 5 |
| 25 | Kidney disease | 2 | Swelling | 3 |
| 26 | Constipation | 2 | Anthrax | 2 |
| 27 | Diabetes | 2 | Evil eye | 2 |
| 28 | Vomiting | 2 | Gadanesa | 2 |
| 29 | Indigestion | 2 | Abdominal pain | 2 |
| 30 | Abortion | 1 | Bloody urine and shivering | 2 |
| 31 | Rabies | 1 | Lelit wof besheta | 2 |
| 32 | Asthema | 1 | Black leg | 1 |
| 33 | Menstrual problem | 1 | Rabies | 1 |
| 34 | Heart disease | 1 | Leech | 2 |
| 35 |  |  | Plazenta delay | 1 |
