# Supplementary material for: Ethnobotanical study of medicinal plants in the Hawassa Zuria District, Sidama zone, Southern Ethiopia
Source: J Ethnobiol Ethnomed. 2019 May 24;15:25. doi: 10.1186/s13002-019-0302-7 (PMC6534827; doi:10.1186/s13002-019-0302-7)
Supplement: Supplementary file 4 — Table S4. Sociodemographic details of the respondents in the Hawassa Zuria district. (DOCX 15 kb) [file 13002_2019_302_MOESM4_ESM.docx]

**Additional file 4:** **Table S4.** Sociodemographic details of the respondents in Hawassa Zuria district.

| **Social group** | **Variables** | **No. of informants (n = 150)** | **Percentage** |
| --- | --- | --- | --- |
| **Gender** | Female | 32 | 21.4 |
|  | Male | 118 | 78.6 |
| **Age** | Young (20–35) | 30 | 20 |
|  | Adult (36–50) | 56 | 37.4 |
|  | Older (>50) | 64 | 42.6 |
| **Education** | Illiterate | 59 | 39.4 |
|  | Basic education | 4 | 2.6 |
|  | Elementary (1–8) | 72 | 48 |
|  | Secondary (9–12) | 12 | 8 |
|  | Tertiary education (10+) | 3 | 2 |
| **Occupation** | Farmer | 114 | 76 |
|  | Herbalist | 20 | 13.4 |
|  | Birth attendants | 7 | 4.6 |
|  | Merchant | 5 | 3.4 |
|  | Student | 4 | 2.6 |
| **Religion** | Protestant | 124 | 83 |
|  | Muslim | 20 | 13 |
|  | None | 6 | 4 |
