# Supplementary material for: Ethnobotanical study of medicinal plants in the Hawassa Zuria District, Sidama zone, Southern Ethiopia
Source: J Ethnobiol Ethnomed. 2019 May 24;15:25. doi: 10.1186/s13002-019-0302-7 (PMC6534827; doi:10.1186/s13002-019-0302-7)
Supplement: Supplementary file 5 — Table S5. Other uses of medicinal plants in the study area. (DOCX 15 kb) [file 13002_2019_302_MOESM5_ESM.docx]

**Additional file 5:** **Table S5.** Other uses of medicinal plants in the study area.

| **Service categories** | **No. of species** | **Percent** |
| --- | --- | --- |
| Food | 20 | 0.33 |
| Fodder | 17 | 0.28 |
| House construction | 16 | 0.26 |
| Firewood | 9 | 0.15 |
| Material cleaning | 6 | 0.08 |
| Robe making | 4 | 0.08 |
| Detergent | 3 | 0.05 |
| Spice | 3 | 0.05 |
| Cash crop | 3 | 0.05 |
| Cultural value | 3 | 0.05 |
| Farming utensils | 2 | 0.03 |
| Tanning | 2 | 0.03 |
| Stimulant | 1 | 0.02 |
| Charcoal | 1 | 0.02 |
| Border division | 1 | 0.02 |
| Antidote for drugs | 1 | 0.02 |
| Fire extinguisher | 1 | 0.02 |
| Fence | 1 | 0.02 |
| Inscense | 1 | 0.02 |
| Furniture | 1 | 0.02 |
| Mattress making | 1 | 0.02 |
| First aid | 1 | 0.02 |
| Hair food | 1 | 0.02 |
| Beverage | 1 | 0.02 |
| Color making | 1 | 0.02 |
| Teeth brushing | 1 | 0.02 |
